# Impact of carotid artery revascularization on cognitive function: a study of symptomatic and asymptomatic cases

**DOI:** 10.3389/fneur.2025.1452495

**Published:** 2025-05-29

**Authors:** Sensen Wu, Yachan Ning, Hui Wang, Dikang Pan, Julong Guo, Cong Wang, Chunmei Wang, Yongquan Gu, Lianrui Guo

**Affiliations:** ^1^Department of Vascular Surgery, Xuanwu Hospital, Capital Medical University, Beijing, China; ^2^Department of Intensive Care Medicine, Xuanwu Hospital, Capital Medical University, Beijing, China

**Keywords:** cognition, carotid artery revascularization, carotid endarterectomy, carotid artery stenting, symptomatic

## Abstract

**Objective:**

To investigate and compare changes in cognitive function following revascularization between patients with symptomatic and asymptomatic carotid stenosis.

**Methods:**

From April 2019 to April 2022, patients carotid artery stenosis who were treated with carotid endarterectomy (CEA) or carotid artery stenting (CAS) were recruited for this study. The Montreal Cognitive Assessment (MoCA) instrument was used to evaluate cognitive function preoperatively and at 3, 6, and 12 months postoperatively. Patients were divided into two groups based on the neurological symptoms, repeated measures ANOVA was used for comparisons.

**Results:**

A total of 89 patients who met the criteria were enrolled and completed 1-year follow-up, divided into symptomatic group (32 patients) and asymptomatic group (57 patients). Baseline data showed no significant differences in clinical characteristics between the two groups. At 3, 6, and 12 months after carotid revascularization, the total MoCA and delayed recall scores for both groups showed significant increases compared to baseline levels. In patients with asymptomatic, attention also showed improvement at 3, 6 months compared to baseline (*p* < 0.05).

**Conclusion:**

Carotid revascularization has a positive impact on cognitive function improvement, particularly in delayed recall and attention.

## Introduction

Carotid artery stenosis is a common atherosclerotic condition and a leading cause of stroke (1, 2). Carotid artery stenosis can be classified as symptomatic or asymptomatic, asymptomatic carotid artery stenosis (ACS) defined as the absence of previous ischemic stroke, transient ischemic attack, or other neurological signs or symptoms (3). To prevent stroke, revascularization procedures such as carotid endarterectomy (CEA) and carotid artery stenting (CAS) are widely employed in clinical practice. Several guidelines have been published to guide clinical management for both symptomatic and asymptomatic carotid artery stenosis (4, 5). The benefits of surgery for symptomatic carotid stenosis are well-established. However, there remains considerable debate regarding the treatment of asymptomatic carotid stenosis, particularly concerning the indications for revascularization procedures.

Cognitive function encompasses various domains, including memory, attention, language abilities, and executive functions, all of which are crucial for maintaining quality of life. Declines in cognitive function can result in impairments across various domains, such as language barriers, decreased computational abilities, weakened judgment, declining memory, visual–spatial dysfunction, and decreased executive function. This reduction in cognitive function reduces work and daily life capabilities, imposing a significant economic burden and psychological stress on families and society (6, 7). Increasing evidence suggests that carotid artery stenosis not only poses a direct threat of stroke but may also impact cognitive function. Patients with carotid artery stenosis may exhibit cognitive decline compared to the normal population, particularly in areas such as attention, executive function, and language abilities (8, 9). These impairments may be associated with chronic cerebral hypoperfusion, leading to white matter lesions, microinfarcts, and brain atrophy (10). In theory, reopening narrowed blood vessels and restoring blood flow to the brain should have beneficial neurocognitive effects. However, previous research findings have been inconsistent, some studies have observed postoperative cognitive improvement, while others have reported deterioration, with results varying among individuals (11–13). The diversity of patient populations, differences in baseline cerebral perfusion status, differences in surgical and endovascular techniques, variation in neuropsychological testing methods, and potential learning effects from repeated testing may lead to this inconsistency. Surgical microembolism, temporary interruption of blood flow, and general anesthesia may also offset the benefits of improving cerebral hemodynamics.

Monitoring changes in cognitive function following carotid revascularization has significant clinical implications. If postoperative cognitive function improves substantially, it can enhance patients’ quality of life and reduce the care burden on families and society. Furthermore, understanding cognitive changes after surgery may help better define the management of asymptomatic carotid stenosis, potentially allowing more patients to benefit from surgical intervention (14). This study aims to compare changes in cognitive function following revascularization between patients with symptomatic and asymptomatic carotid stenosis. By systematically evaluating cognitive function at different time points before and after surgery, we seek to analyze cognitive changes post-revascularization in patients with carotid stenosis. This can aid in developing more precise surgical strategies, minimizing surgical risks, and optimizing treatment outcomes.

## Study subjects and methods

### Study subjects

This study is a single-center, prospective observational study. We selected patients with severe carotid stenosis who were hospitalized in our department and scheduled for carotid endarterectomy (CEA) or carotid artery stenting (CAS) between 2019 and 2022. The study was approved by our local Ethics Committee and was conducted in accordance with the Helsinki Declaration. All participants or their legal representatives provided informed consent.

### Inclusion criteria

(1) Age ≥ 40 years; (2) Diagnosis of at least unilateral carotid stenosis of ≥ 70% confirmed by at least two diagnostic methods: non-invasive methods such as color Doppler carotid ultrasound, computed tomography angiography (CTA), magnetic resonance angiography (MRA), and invasive digital subtraction angiography (DSA); (3) Patients scheduled for CEA or CAS; (4) Ability to independently complete cognitive function assessments.

### Exclusion criteria

(1) Refusal to undergo cognitive function assessments or other relevant examinations; (2) Refusal to comply with follow-up; (3) History of depression or psychiatric disorders; (4) Known causes of cognitive impairment, such as Parkinson’s disease, Alzheimer’s disease, epilepsy, etc.; (5) Presence of aphasia, dysarthria, visual, or hearing impairments; (6) Previous CEA or CAS; (7) Severe systemic diseases or major organ failure; (8) Concurrent malignant tumors; (9) Life expectancy of less than 1 year.

### Data collection

Patients meeting the inclusion criteria underwent detailed history taking, physical examination, and collection of general data and baseline characteristics. This included general information (age, gender, years of education, hypertension, hyperlipidemia, diabetes, coronary artery disease, atrial fibrillation, smoking history, alcohol consumption, white matter degeneration, lesion location, plaque characteristics, stenosis degree), laboratory tests (blood routine, liver and kidney function, blood lipids, muscle enzyme spectrum, C-reactive protein, electrolytes, coagulation function), cognitive function assessments using evaluation scales, and imaging examinations (vascular ultrasound, cranial magnetic resonance imaging(MRI) with diffusion-weighted imaging(DWI), cranial computed tomography). Perioperative complications and special events were also recorded. All patients underwent clinical, cognitive assessment, and imaging examinations within 1 week before the carotid revascularization. MRI with DWI sequence was performed before carotid revascularization and within 1 week after the procedure to determine subclinical microembolism, which did not cause symptoms and positive signs of neurological impairment.

### Cognitive function assessment

Cognitive function was assessed using the Chinese version of the Montreal Cognitive Assessment (MoCA) (15, 16). MoCA includes 10 cognitive domains: trail making test, cube copy, clock drawing, naming, attention, sentence repetition, verbal fluency, abstraction, delayed recall, and orientation. The maximum score on the MoCA is 30 points, with a score of ≥ 26 considered normal cognitive function and a score < 26 indicating cognitive impairment. Specific MoCA domain scores are as follows: visuospatial/executive (5 points) assessed by trail making test (1 point), cube copy (1 point), and clock drawing (3 points). Naming test involves identifying animals (3 points). Attention (6 points) is assessed by sustained attention task (1 point), continuous subtraction (3 points), and forward and backward digit span (1 point each). Language (3 points) is assessed by repeating two syntactically complex sentences (2 points) and verbal fluency test (1 point). Abstraction (2 points) is assessed using similarity tasks. Short-term delayed recall (5 points) involves learning and recalling five words after approximately 5 min. Finally, orientation (6 points) assesses time and place. Patients with ≤ 12 years of education receive an additional point to correct for educational bias. All cognitive assessments were conducted by trained and certified physicians (17).

### Carotid revascularization

Carotid revascularization procedures were performed by trained vascular surgeons. The choice of revascularization method was determined by the vascular surgeon and was not influenced by this study. Patients received aspirin (100 mg/day) for at least 3 days before CEA or a combination of aspirin (100 mg/day) and clopidogrel (75 mg/day) for at least 3 days before CAS. CEA was performed under general anesthesia with continuous intraoperative transcranial Doppler monitoring. CAS was performed under local anesthesia via the femoral artery with continuous ECG monitoring. A self-expanding stent (Wallstent) was placed at the stenotic site with distal embolic protection. The stent diameter and post-dilation were determined by the surgeon. All patients received at least 24 h of intensive care postoperatively and continued aspirin (100 mg/day) and clopidogrel (75 mg/day) for 3 months, followed by long-term aspirin therapy alone.

### Follow-up

All patients were followed up at 3, 6, and 12 months post-revascularization via outpatient visits, during which cognitive assessments were performed by professional doctors. The primary endpoints included changes in MoCA scores at 3, 6, and 12 months postoperatively. Secondary endpoints included complications within 30 days postoperatively and the incidence of transient ischemic attacks, ischemic strokes, or other cardiovascular events within 12 months.

### Statistical analysis

Statistical analysis was conducted using SPSS 26.0 and R (version 4.2.2). Continuous variables with normal distribution were presented as mean ± standard deviation (SD), while non-normally distributed variables were presented as median and interquartile range (IQR). Categorical variables were expressed as frequencies and percentages. Comparisons of categorical variables were performed using Chi-square or Fisher’s exact tests. Continuous variables were compared using Welch’s Two-Sample t-test. Repeated Measures ANOVA was used for intra-group and inter-group comparisons, with Mauchly’s test of sphericity used to test for sphericity. If sphericity was violated, Huynh-Feldt correction was applied. *Post hoc* tests were conducted using Tukey’s Honestly Significant Difference when *p* < 0.05. A *p*-value < 0.05 was considered statistically significant.

## Results

### Baseline characteristics of patients

A total of 89 patients participated in the study, divided into a symptomatic group (32 patients) and an asymptomatic group (57 patients). Baseline data showed no significant differences in clinical characteristics between the two groups (Table 1). Specifically, the average age of participants was 66.94 years, with a higher proportion of male patients (82.02%). There were no significant differences in baseline characteristics such as years of education and body mass index (BMI). Regarding comorbidities, the prevalence of coronary artery disease, hypertension, diabetes, hyperlipidemia, smoking history, and alcohol consumption were comparable between the two groups. These baseline data indicate that the two groups had similar health status characteristics before surgery, providing a solid foundation for subsequent comparative analyses.

**Table 1:** Basic information of patients.

Characteristic	Total (*n* = 89)	Symptomatic (*n* = 32)	Symptomatic (*n* = 57)	*p*-value*
Demographic and clinical characteristics				
Age, year, Mean ± SD	66.94 ± 8.53	68.22 ± 8.28	66.23 ± 8.66	0.293
Male, n(%)	73 (82.02)	26 (81.25)	47 (82.46)	0.887
Education, year, Mean ± SD	9.24 ± 3.75	9.91 ± 3.96	8.86 ± 3.61	0.209
BMI, Mean ± SD	24.97 ± 2.78	25.16 ± 3.02	24.87 ± 2.66	0.646
Side, n(%)	39 (43.82)	12 (37.50)	27 (47.37)	0.368
CAD, n(%)	28 (31.46)	9 (28.12)	19 (33.33)	0.612
Hypertension, n(%)	68 (76.40)	23 (71.88)	45 (78.95)	0.451
Diabetes, n(%)	35 (39.33)	16 (50.00)	19 (33.33)	0.122
Hyperlipidemia, n(%)	43 (48.31)	15 (46.88)	28 (49.12)	0.839
Smoking history, n(%)	45 (50.56)	15 (46.88)	30 (52.63)	0.602
Alcohol consumption, n(%)	36 (40.45)	12 (37.50)	24 (42.11)	0.671
White matter lesions, n(%)	44 (49.44)	17 (53.12)	27 (47.37)	0.602
Ipsilateral vertebral artery stenosis, n(%)	10 (11.24)	4 (12.50)	6 (10.53)	1.000
Ulcerative plaques, n(%)	6 (6.74)	4 (10.26)	2 (4.00)	0.398
Contralateral carotid artery stenosis, n(%)	9 (10.11)	4 (12.50)	5 (8.77)	0.847
Contralateral vertebral artery stenosis, n(%)	12 (13.48)	4 (12.50)	8 (14.04)	1.000
Perioperative and follow-up outcomes				
Surgical techniques, n(%)				0.122
CEA	54 (60.67)	16 (50.00)	38 (66.67)	
CAS	35 (39.33)	16 (50.00)	19 (33.33)	
Hypotension, n(%)	4 (4.49)	1 (3.12)	3 (5.26)	1.000
Bradycardia, n(%)	7 (7.87)	2 (6.25)	5 (8.77)	0.989
Hematoma, n(%)	1 (1.12)	0 (0.00)	1 (1.75)	1.000
Nerve injury, n(%)	5 (5.62)	1 (3.12)	4 (7.02)	0.775
New infarcted lesions in MRI, n(%)	8 (8.99)	3 (9.38)	5 (8.77)	1.000
Stroke	2 (2.25)	1 (3.12)	1 (1.75)	1.000
Restenosis	5 (5.62)	2 (6.25)	3 (5.26)	1.000

*Welch Two Sample t-test; Pearson’s Chi-squared test; Fisher’s exact test. BMI, Body mass index; CAD, Coronary artery disease; SD, standard deviation; CEA, carotid endarterectomy; CAS, carotid artery stenting; MRI, Magnetic resonance imaging.

### Changes in cognitive function

Changes in cognitive function were the primary outcomes observed in this study. Analysis of cognitive test results at different time points revealed significant improvements in postoperative cognitive function for both symptomatic and asymptomatic patients (Table 2). MoCA scores for both groups showed significant increases at 3, 6, and 12 months postoperatively compared to baseline levels. The symptomatic group improved from a baseline score of 22.31 to 24.00 at 12 months postoperatively, while the asymptomatic group improved from 22.19 to 23.86. Additionally, in the delayed recall test, the symptomatic group improved from a baseline score of 1.75 to 2.88 at 12 months postoperatively, and the asymptomatic group improved from 1.98 to 3.11, indicating a significant enhancement in memory function post-surgery. Attention test results also showed a similar trend, with scores improving in both the symptomatic and asymptomatic groups.

**Table 2:** Effect of revascularization on cognition among all patients.

Cognition test	Baseline		3 months		6 months		12 months	
	Symptomatic	Asymptomatic	Symptomatic	Asymptomatic	Symptomatic	Asymptomatic	Symptomatic	Asymptomatic
MoCA score	22.31 ± 4.21^a^	22.19 ± 3.81^a^	23.56 ± 4.48^a^	23.44 ± 3.78^a^^,^^b^	23.94 ± 4.32^a^	23.89 ± 3.49^a^^,^^b^	24.00 ± 4.40^a^	23.86 ± 3.70^a^^,^^b^
Visuospatial / executive	3.22 ± 1.41	3.16 ± 1.32	3.37 ± 1.38	3.23 ± 1.28	3.44 ± 1.37	3.26 ± 1.22	3.50 ± 1.39	3.32 ± 1.21
Naming	2.91 ± 0.30	2.67 ± 0.66	2.88 ± 0.42	2.75 ± 0.58	2.91 ± 0.30	2.75 ± 0.58	2.91 ± 0.30	2.77 ± 0.57
Attention	4.91 ± 1.44^a^^,^^c^	5.07 ± 1.08^a^^,^^c^	5.16 ± 1.27	5.19 ± 1.01^a^	5.19 ± 1.23	5.30 ± 0.96^a^	5.16 ± 1.22	5.26 ± 1.01
Language	1.50 ± 0.92	1.47 ± 0.89	1.59 ± 1.01	1.58 ± 0.92	1.66 ± 1.07	1.61 ± 0.88	1.66 ± 1.07	1.67 ± 0.89
Abstraction	1.59 ± 0.67	1.28 ± 0.70	1.59 ± 0.67	1.37 ± 0.70	1.59 ± 0.67	1.39 ± 0.67	1.56 ± 0.67	1.35 ± 0.67
Delayed recall	1.75 ± 1.32^a^	1.98 ± 1.34^a^	2.41 ± 1.64^a^	2.70 ± 1.56^a^^,^^b^	2.78 ± 1.56^a^	3.02 ± 1.38^a^^,^^b^	2.88 ± 1.34^a^	3.11 ± 1.36^a^^,^^b^
Orientation	5.66 ± 0.55	5.63 ± 0.72	5.81 ± 0.40	5.74 ± 0.52	5.75 ± 0.44	5.70 ± 0.63	5.75 ± 0.44	5.72 ± 0.56

a *p* < 0.05, Comparison of results between postoperative and preoperative.

b *p* < 0.05, Comparison of results between postoperative 6 months or 12 months and 3 months.

c *p* < 0.05, Comparison of the baseline data between-group.

### Perioperative and follow-up outcomes

There were no significant differences in the incidence of postoperative complications between the two groups (Table 1). Regarding the choice of surgical method, 54 patients underwent CEA, and 35 patients underwent CAS, with no significant difference in the choice of surgery between the symptomatic and asymptomatic groups. The overall incidence of hypotension was 4.49%, and the incidence of new MRI lesions was 8.99%. The incidence of symptomatic stroke events was 2.25%, with one case in each group. The rate of restenosis (>50%) during the 12-month follow-up was 5.62%, with no significant difference between the two groups. These results suggest that carotid revascularization is associated with high perioperative and follow-up safety and efficacy.

## Discussion

Our results demonstrate significant improvements in cognitive function following carotid revascularization in both symptomatic and asymptomatic patients, with MoCA scores steadily increasing at 3, 6, and 12 months postoperatively. Notably, delayed recall and attention showed substantial enhancement, particularly in asymptomatic patients, suggesting potential cognitive benefits beyond stroke prevention. Importantly, the absence of significant baseline differences between the two groups strengthens the reliability of our findings. Furthermore, our study confirms the safety and efficacy of carotid revascularization, with low rates of perioperative complications, including hypotension (4.49%), new MRI lesions (8.99%), and symptomatic stroke events (2.25%), as well as a low restenosis rate (5.62%) at 12 months. These findings support the role of carotid revascularization not only in reducing cerebrovascular risk but also in preserving cognitive function, emphasizing the importance of timely intervention.

In the early 1950s, Fisher (18) first proposed that carotid artery disease and hemodynamic disturbances could impact cognitive function. The Tromsø study (19) further demonstrated that subjects with carotid artery stenosis performed significantly lower in several subtests of cognitive assessments. Recent studies suggest that ACS is also associated with cognitive impairment. Approximately 49.4% of patients exhibit deficits in at least two neuropsychological domains, indicating that asymptomatic carotid artery stenosis may clinically impact cognitive decline and dementia risk more than it contributes to stroke risk (20). The CREST-2 (Carotid Revascularization and Medical Management for Asymptomatic Carotid Stenosis Trial) (21) study recently conducted two trials on primary stroke prevention in patients with severe asymptomatic carotid artery stenosis. The trials aimed to compare the best management strategies for asymptomatic carotid artery stenosis and explore whether cognitive abilities are compromised before treatment in asymptomatic carotid disease. Ronald M. Lazar (22) and colleagues studied the cognitive status of 1,000 consecutive CREST-2 patients and found that, after controlling for demographic and cardiovascular risk factors, patients with severe carotid artery stenosis had lower baseline cognitive function than those with normal cognition. This cohort represents the largest group to date demonstrating poorer cognitive abilities, particularly memory, in patients with this condition. These studies suggest a correlation between carotid artery stenosis and cognitive impairment, regardless of the presence of neurological symptoms. A recent systematic review (23) concluded that severe ACS is associated with progressive declines in multiple aspects of cognitive function, including overall cognition, memory, and executive function. Patients with ACS are at an increased risk of cognitive decline, and evidence from this review indicates that classifying ACS patients as “asymptomatic” may be inappropriate given their cognitive impairment. Future research should include cognitive assessments as part of the outcomes, alongside stroke, myocardial infarction, and mortality rates.

Recent evidence has suggested that cerebral hypoperfusion is an important cause of cognitive impairment (24). The mechanisms through which severe carotid artery stenosis affects cognitive function remain incompletely understood, as cognitive function is influenced by multiple factors. One significant pathway involves reduced cerebral perfusion: while mild to moderate stenosis typically does not compromise cerebral blood flow due to the brain’s autoregulation, severe stenosis can markedly impair perfusion. This reduction in blood flow deprives neuronal tissues of oxygen and nutrients, triggering oxidative stress and neuronal damage, which manifest as cognitive deficits (25). Another mechanism is asymptomatic microembolism (20), where plaque fragments dislodged from the carotid artery can cause small emboli. These microemboli may affect brain regions supplied by the carotid artery, such as the frontal and temporal lobes, hippocampus, and limbic system, potentially leading to subtle cognitive impairments that are not clinically evident. Furthermore, carotid artery stenosis is associated with increased white matter lesions in the brain (26). These lesions disrupt neural networks and communication between brain regions, further contributing to cognitive dysfunction. Understanding these pathways is crucial for developing targeted interventions aimed at preserving cognitive function in individuals with carotid artery stenosis. Further research is needed to explore these mechanisms comprehensively and to develop effective therapeutic strategies.

Current research on cognitive function changes following carotid revascularization procedures presents some controversies and gaps. Firstly, the results regarding postoperative cognitive function are inconsistent; some studies report cognitive improvement, while others indicate cognitive decline (11–13). Secondly, the specific mechanisms underlying these cognitive changes remain unclear, with individual patient differences potentially leading to varied outcomes. Moreover, most studies focus on short-term follow-ups, leaving a relative paucity of data on long-term cognitive changes post-surgery (27). In this study, we comprehensively assessed cognitive function changes in symptomatic and asymptomatic patients with carotid artery stenosis undergoing revascularization procedures, including CEA and CAS. By grouping patients based on symptoms and ensuring no significant differences in baseline clinical characteristics between the groups, we could fairly compare postoperative cognitive changes while minimizing confounding factors. Overall, our analysis of cognitive test results at various time points demonstrates that carotid revascularization positively impacts cognitive function in both symptomatic and asymptomatic carotid stenosis patients. Postoperative MoCA scores, delayed recall, and attention metrics significantly improved, with some improvements persisting up to 12 months post-surgery. Additionally, revascularization procedures showed high safety and efficacy during the perioperative period and follow-up, without significantly increasing the risk of complications.

The efficacy of carotid revascularization in the prevention of symptomatic carotid stenosis stroke has been widely validated, and active surgical intervention is recommended (4, 5). However, its effectiveness in preventing cognitive impairment in asymptomatic carotid stenosis remains contentious (23). Current evidence suggests that revascularization may improve cognitive function by restoring cerebral blood flow, correcting chronic cerebral hypoperfusion, and reducing microemboli formation (24). Multiple previous studies have analyzed the cognitive function changes post-carotid revascularization in both symptomatic or asymptomatic patients, with most indicating improvements in cognitive function post-surgery. A study on symptomatic patients with severe carotid stenosis showed improvements in MoCA score and attention within the first 12 months post-revascularization, and the drug-control group had significantly decreased cognition (28). Similarly, elderly patients with severe symptomatic stenosis exhibited enhanced cognitive abilities, including MoCA and MMSE scores, post-CEA (29). Usman (30) also confirmed the efficacy of CEA for ACS cognitive improvement, for patients included in ACS in CEA before, 4 weeks, 12 weeks after Addenbrookes cognitive score scale (ACE) and general practitioner cognitive assessment scale (GPCOG), the results show that both scales improved before and after surgery. Nonetheless, several studies have reported postoperative cognitive decline, possibly linked to intraoperative microembolization signals and the emergence of new intracranial microinfarcts visible on postoperative MRI (26, 31, 32).

Our findings align with these many results, showing no significant differences in baseline MoCA scores and sub-items between the two groups. Notably, the asymptomatic group demonstrated better improvements in sentence repetition at 6 and 12 months compared to the symptomatic group. Considering the potential impacts of intraoperative hypoperfusion, microembolism, general anesthesia, and postoperative hyperperfusion, the relationship between carotid revascularization and cognitive function remains complex and multifaceted. Further research, particularly long-term follow-up studies, is necessary to elucidate these mechanisms and clarify the cognitive outcomes associated with these procedures. Current evidence-based medicine highlights the need for ongoing investigation to untangle the intricate connections between carotid revascularization and cognitive function.

There is a great controversy about whether patients with asymptomatic carotid artery stenosis should be treated surgically. In addition to this goal beyond stroke prevention, minimizing cognitive decline in asymptomatic and symptomatic carotid patients may be a new research direction and target for carotid revascularization. Although there is no complete consensus among the investigators on the effects of revascularization on cognitive function, it seems to emphasize the beneficial effects of carotid revascularization on cognitive function. In our study, we compared the changes in cognitive function in symptomatic and asymptomatic patients during the 1-year follow-up period of surgery and systematically evaluated the effect of revascularization on their cognitive function. The baseline level of the two groups, only the attention score is higher in asymptomatic patients, the results showed that carotid revascularization can improve the cognitive function of patients, and more improvement items in asymptomatic patients, supporting the potential benefits of surgery in improving cognitive function, which has important guiding significance for the choice of treatment strategies in patients with asymptomatic carotid stenosis. Given the low complication rate of surgery and the significant cognitive improvement effect, perhaps in the future cognitive function could be considered as an additional indication for carotid revascularization. To enhance the robustness of future studies, further research will require more suitable, comprehensive and uniform neurocognitive assessment tools, as well as more advanced comprehensive imaging techniques, to identify patient populations that benefit from carotid reconstruction. Additionally, a larger sample size should be included to validate these findings. Investigating the specific mechanisms of cognitive improvement, particularly regarding cerebral blood flow, brain metabolism, and neural network remodeling, will provide further insight into the underlying processes. Moreover, extending the follow-up period beyond 12 months is crucial for assessing long-term cognitive changes and the long-term safety and efficacy of carotid revascularization surgery.

## Limitations

Despite the significant findings of this study, there are several limitations. Firstly, the sample size is relatively small, which may affect the generalizability and reliability of the results. Secondly, this study primarily relies on cognitive test results to assess postoperative cognitive changes. Cognitive function is a complex, multidimensional concept, and a single cognitive test may not comprehensively reflect the patients’ cognitive status, in our study, cognitive function was assessed solely using the MoCA, a screening tool designed to detect mild cognitive impairment. While the MoCA provides a rapid and standardized measure of global cognition, it may lack sensitivity to evaluate specific cognitive domains (e.g., executive function, visuospatial memory) in depth, incorporating additional neuropsychological tests [e.g., ey-Osterrieth Complex Figure Test (ROCF) and the Wisconsin Card Sorting Test (WCST)] could strengthen the validity of conclusions regarding domain-specific cognitive changes. Additionally, this study lacks objective measures to explore the relationship between blood flow changes after revascularization and cognitive function. Lastly, the follow-up period is limited to 12 months, which may not be sufficient to evaluate long-term cognitive changes and the lasting effects of the surgery.

## Conclusion

This study systematically evaluated the cognitive function of symptomatic and asymptomatic carotid stenosis patients post-revascularization, revealing that carotid revascularization has a positive impact on cognitive function improvement, particularly in delayed recall and attention. These findings provide critical evidence for clinical practice, supporting carotid revascularization as an effective treatment for carotid stenosis, with potential cognitive benefits. However, further research is necessary to confirm these findings, explore the underlying mechanisms, and assess the long-term effects.

## Data Availability

The raw data supporting the conclusions of this article will be made available by the authors, without undue reservation.
